# Nanoscale analysis of superconducting Fe(Se,Te) epitaxial thin films and relationship with pinning properties

**DOI:** 10.1038/s41598-021-99574-5

**Published:** 2021-10-11

**Authors:** Mario Scuderi, Ilaria Pallecchi, Antonio Leo, Angela Nigro, Gaia Grimaldi, Carlo Ferdeghini, Corrado Spinella, Marco Guidolin, Antonio Trotta, Valeria Braccini

**Affiliations:** 1 CNR – IMM Catania Headquarter, Strada VIII n.5 Zona Industriale, 95121 Catania, Italy; 2 CNR – SPIN Genova, Corso Perrone n.24, 16152 Genova, Italy; 3 CNR – SPIN Salerno, Via Giovanni Paolo II n.132, 84084 Fisciano, Salerno Italy; 4 Dipartimento di Fisica “E.R. Caianiello”, Università Di Salerno, Via Giovanni Paolo II n.132, 84084 Fisciano, Salerno Italy; 5 Eni Venezia, Via delle Industrie n.39, 30175 Marghera, Italy

**Keywords:** Superconducting properties and materials, Characterization and analytical techniques, Transmission electron microscopy

## Abstract

The process of developing superconducting materials for large scale applications is mainly oriented to optimize flux pinning and the current carrying capability. A powerful approach to investigate pinning properties is to combine high resolution imaging with transport measurements as a function of the magnetic field orientation, supported by a pinning modelling. We carry out Transmission Electron Microscopy, Electron Energy Loss Spectroscopy and critical current measurements in fields up to 16 T varying the angle between the field and *c*-axis of Fe(Se,Te) epitaxial thin films deposited on CaF_2_ substrates. We find evidence of nanoscale domains with different Te:Se stoichiometry and/or rotated and tilted axes, as well as of lattice distortions and two-dimensional defects at the grain boundaries. These elongated domains are tens of nm in size along the in-plane axes. We establish a correlation between these observed microstructural features and the pinning properties, specifically strongly enhanced pinning for the magnetic field oriented in-plane and pinning emerging at higher fields for out-of-plane direction. These features can be accounted for within a model where pinning centers are local variations of the critical temperature and local variations of the mean free path, respectively. The identification of all these growth induced defects acting as effective pinning centers may provide useful information for the optimization of Fe(Se,Te) coated conductors.

## Introduction

Fe(Se,Te) superconducting compounds are of huge interest from the application point of view, due to their high upper critical field and the possibility of deposition on technical substrates for the fabrication of coated conductors with excellent critical current performances. Most important, Fe(Se,Te)-based coated conductors can be fabricated with less stringent in-plane texturing requirements than REBCO-based coated conductors, thanks to the weaker suppression of inter-granular critical current with misalignment angle between adjacent grains^1^, and also with relatively lower fabrication costs. Fe(Se,Te) coated conductors can be successfully grown on RABiTS^2–4^ and IBAD^5^ substrates already developd for REBCO, as well as on simpler templates such as HASTELLOY C276 with native oxide^4^, Mica^6^, amorphous glass^7^ or stainless steel with amorphous Al_2_O_3_ layer^8^. For Fe(Se,Te) coated conductors, self-field critical current values, J_c_, above 10^6^ A/cm^2^ at 4.2 K, and still as high as 10^5^ A/cm^2^ at 30 T magnetic fields have been reported^2^. Technical substrates in REBCO coated conductors are complex architectures with multiple layers, including also epitaxial oxide layers, whose primary role is favoring texturing. However, the stacking of oxide layers also causes growth induced defects, which ultimately determine the pinning properties of the superconducting film. Hence, with the coated conductor application in mind, the study of the superconducting and pinning properties of Fe(Se,Te) films deposited on different crystalline substrates may provide precious information for performances optimization of the coated conductors.

Generally, films deposited on CaF_2_ single crystals exhibit larger and more isotropic J_c_ than films deposited on SrTiO_3_ or other substrates. In Ref.^9^, a self-field J_c_ above 10^6^ A/cm^2^ was reported at 4.2 K for a film deposited on CaF_2_; J_c_^par^ decreased to 4.5 × 10^5^ A/cm^2^ in field of 9 T with a very low anisotropy ratio J_c_^par^/J_c_^perp^ of about 1.07. For comparison, in a film deposited on SrTiO_3_, J_c_^par^ at 4.2 K and 9 T was 7.3 × 10^4^ A/cm^2^, with an anisotropy ratio J_c_^par^/J_c_^perp^ ≈ 0.67. Given that critical temperature values, T_c_ were comparable for the two films deposited on CaF_2_ and SrTiO_3_, and that there is no systematic trend of higher or lower upper critical field values, H_c2,_ for films deposited on CaF_2_ in comparison with films deposited on SrTiO_3_, the larger self-field and in-field J_c_’s in the films on CaF_2_ suggests a role of pinning centers induced by the growth on CaF_2_. Even more important, the isotropic character of J_c_ in films on CaF_2_ indicates that these growth-induced pinning centers are effective for all the directions of the flux lines. Critical current anisotropy J_c_(θ,H,T) was deeply studied in these films deposited on CaF_2_^10^ to investigate the high current capability of this material for high fields applications in comparison with the competitors High Temperature Superconductors (HTS). In Ref.^11^, the field dependence of the activation energy for flux line motion in films deposited on CaF_2_ was described as a power law, with an exponent that varied in different ranges of field. This indicates that the pinning regime varies with field, likely due to the existence of different types of pinning mechanisms with different strength for the field applied parallel or perpendicular to the *ab* planes.

The above results on different pinning properties of Fe(Se,Te) films deposited on SrTiO_3_ and CaF_2_ gave us the motivation to investigate more in depth the pinning mechanisms in Fe(Se,Te) films grown on CaF_2_, in terms of structure, microstructure, stoichiometry and superconducting properties. In this work, we use a combined approach of imaging, elemental mapping, electrical transport measurements and modelling to carry out such deep investigation on thin films grown on CaF_2_ through Pulsed Laser Deposition. Films are about 100 nm thick and show T_c_ of about 17 K and resistivity in the normal state (at 20 K) of about 200 µΩ cm. We present Scanning Transmission Electron Microscopy (STEM), Electron Energy Loss Spectroscopy (EELS) and electrical transport J_c_ measurements in fields up to 16 T and as a function of the angle between the applied field and the *c*-axis, and we correlate superconducting properties with microstructural defects and local stoichiometry inhomogeneity.

## Results

### Nanostructural analysis

**Figure 1:**
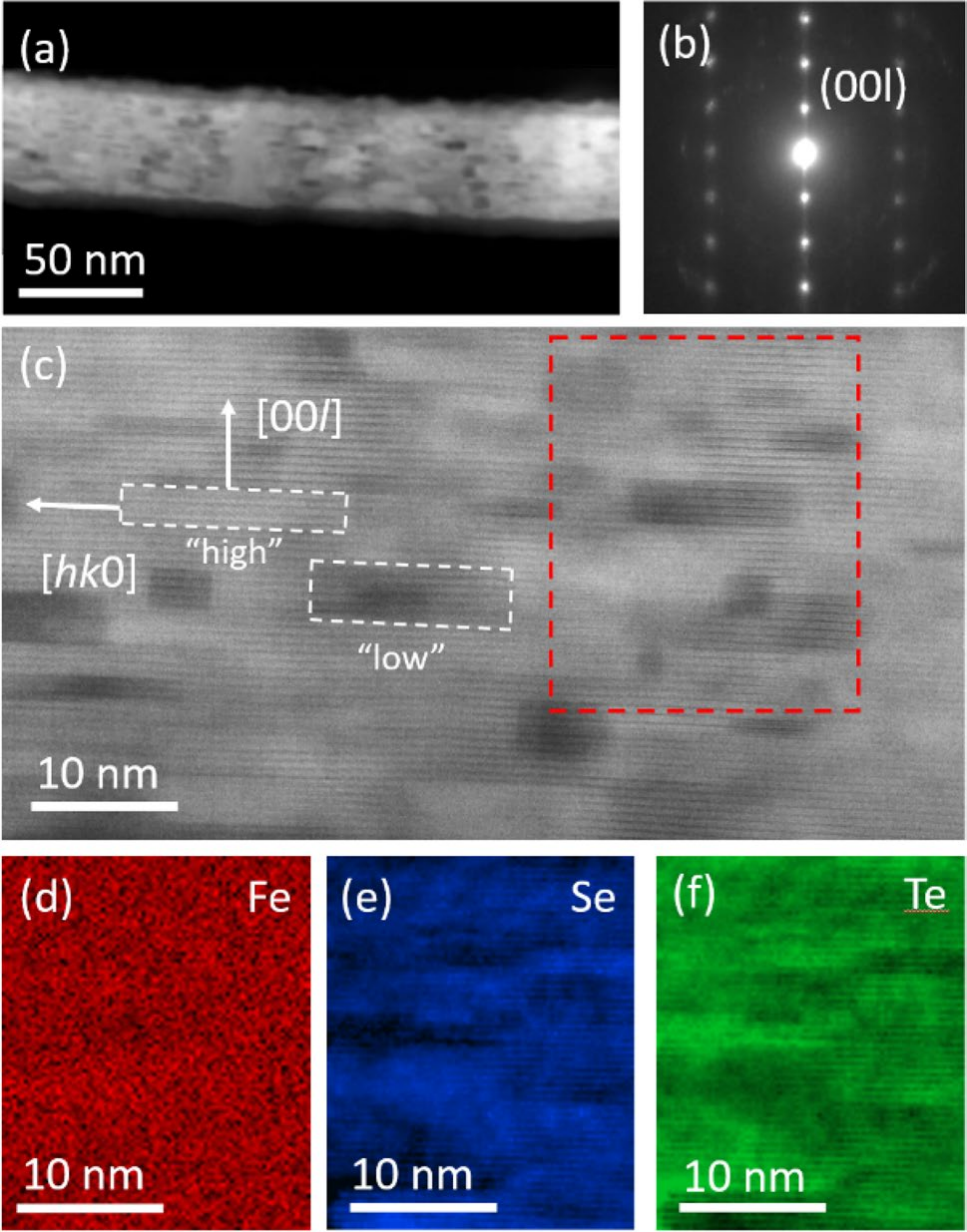
Electron microscopy images and elemental maps of the Fe(Se,Te) thin-film. (**a**) STEM cross-sectional image. (**b**) Corresponding SAED pattern. (**c**) Enlarged view of the Fe(Se,Te) film indicating high- and low-atomic mass grains. (**d**), (**e**) and (**f**) Corresponding EELS elemental maps of the portion indicated by a red dashed line in panel (**c**).

Both cross- and plane-view Transmission Electron Microscopy (TEM) analyses were performed on the Fe(Se,Te) film in order to collect structural information from both in-plane and out-of-plane view, i.e. along the axis [110] and [001], respectively. A cross-sectional view of the Fe(Se,Te) film is shown in Fig. 1a. In this micrograph a portion of the Fe(Se,Te) film exfoliated from the CaF_2_ substrate and embedded in epoxy, is shown. The sharp out-of-plane (00*l*) reflections from Selected Area Electron Diffraction (SAED) data (Fig. 1b) show that the film exhibits a high degree of epitaxy and the predominant in-plane texture orientation was found to be [110]Fe(Se,Te) II [100] CaF_2_ (b spot of SAED diffraction).

On the micrographs of Fig. 1, the experimental conditions were properly chosen to attain a pure Z-contrast, minimizing the contribution from diffraction contrast^12^. Thus, the observed contrast can be ascribed to a different average atomic mass, suggesting the presence of clusters with different stoichiometry, as also confirmed by EELS elemental maps described hereafter. In detail, on the enlarged view of a film portion shown in Fig. 1c, bright and dark regions mark out the shape of some grains with high- and low-atomic mass respectively.

 Because of projection effects due to TEM technique causing multiple grain overlapping along the thickness of the sample, the elongated shape of the grains is well recognizable only for dark grains (low effective atomic mass), while in the other cases the superposition of various grains makes it difficult to recognize the grain profile. Moreover, by looking at Fig. 1c it is evident that it is easier to recognize the horizontal interfaces of grains than the vertical ones. Precisely, in the image in Fig. 1c we can notice that boundaries along the horizontal direction ((00*l*) planes) are more apparent than those along the vertical ones ((*hk*0) planes). This means that the (00*l*) grain boundaries are well aligned with the electron beam ([110] Fe(Se,Te) direction), while (*hk*0) boundaries are not. As a whole, the grains show a flattened shape with the smallest dimension along the *c*-axis. The typical grain is 15–20 nm wide and 3–5 nm high.

EELS spectra were acquired from a representative portion of the film that includes grains with different contrast (red dashed line of Fig. 1c) and elemental maps were obtained by selecting signal from Fe L-, Se L- and Te M-edge on Fig. 1d–f respectively. The maps confirm the presence of Te-rich grains, where the corresponding Z-contrast image shows bright regions. The Se and Te contents are complementary since they occupy the same lattice site, while the Fe content is quite uniform.

**Figure 2:**
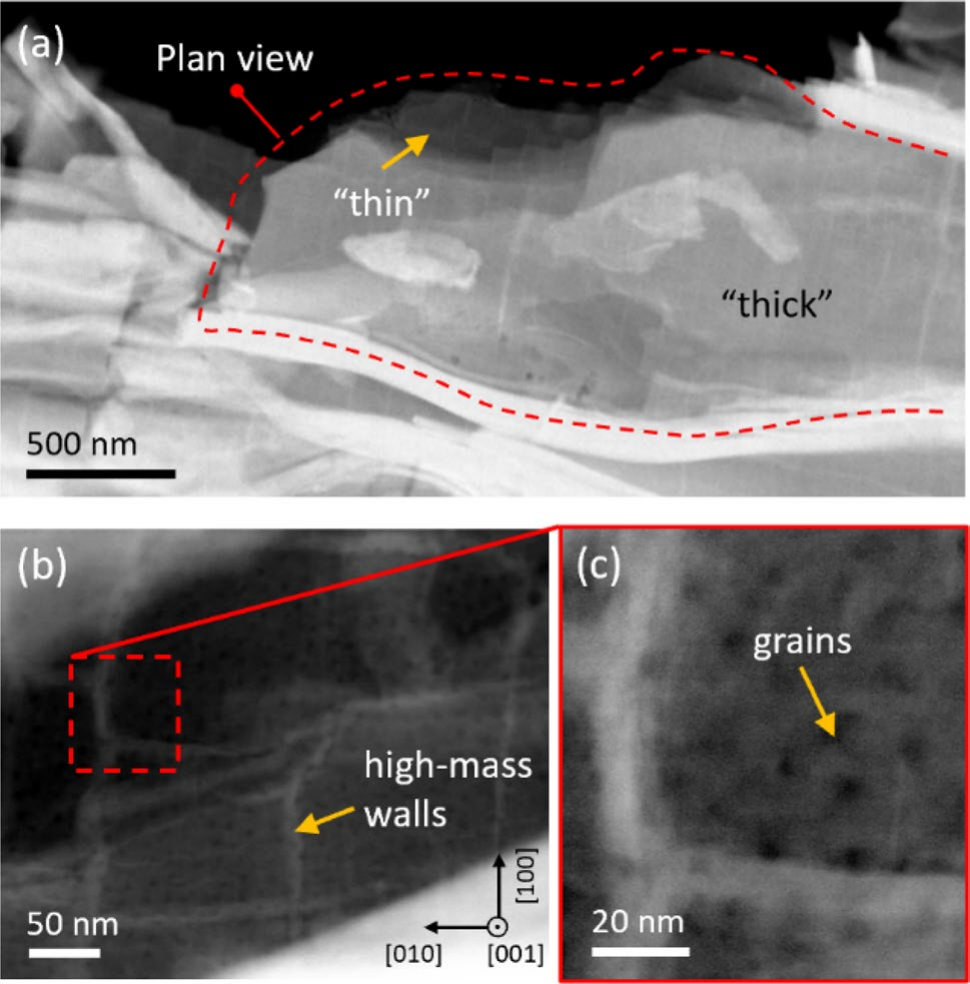
STEM micrographs of the Fe(Se,Te) film detached from the CaF_2_ substrate. (**a**) Low-magnification of the crumbled film with a red dashed line indicating the portion that is nearly in-plane; (**b**) detail of the plane-view showing high-mass walls; (**c**) detail showing the grain structure of the film (**c**).

We complete the structural information collected so far by adding a top-view STEM analysis of the film. In Fig. 2a, a freestanding portion of the film exfoliated from the CaF_2_ substrate is shown. Here the film is crumpled due to the mechanical process used to transfer it onto the TEM grid, thus, in this micrograph, the film appears as randomly oriented and offers different orientations in the different regions of the image. In particular, we get a plane view of the film in the central part of the figure indicated by a red-dotted line. Here, in the portion labelled as “thick”, the film is typically 50 nm thick. Given that the grains about 3–5 nm high, as obtained by cross-section measurements in Fig. 1, we conclude that in this region the film is composed by several piled-up grains. In the top portion labelled as “thin”, the film gets thinner reaching approximately the thickness of 10 nm, as measured by applying the log-ratio method to the EELS spectra in this region. Thus, we can assert that overlapping between different grains in this region is strongly reduced and the film is almost one-grain thick.

Figure 2b, c show two higher magnification micrographs of the film taken on the “one-grain thick” region. Both images show that along the *ab* plane the film exhibits some structures. From Fig. 2b, we evidence the presence of bright filaments oriented along the {100} equivalent planes. For a Z-contrast image, these filaments indicate a high effective atomic mass, i.e. a compositional variation, also confirmed by EELS analysis (see Supplementary Information). Specifically, these filaments are Se/Te-rich structures that roughly define square-like domains around 120 nm per side. Hereafter we call these structures high-mass walls. In Fig. 2c the grain structure observed in plane-view emerges. The typical scale of these structures is 20–30 nm, as measured by both plane- and cross-sectional analyses.

**Figure 3:**
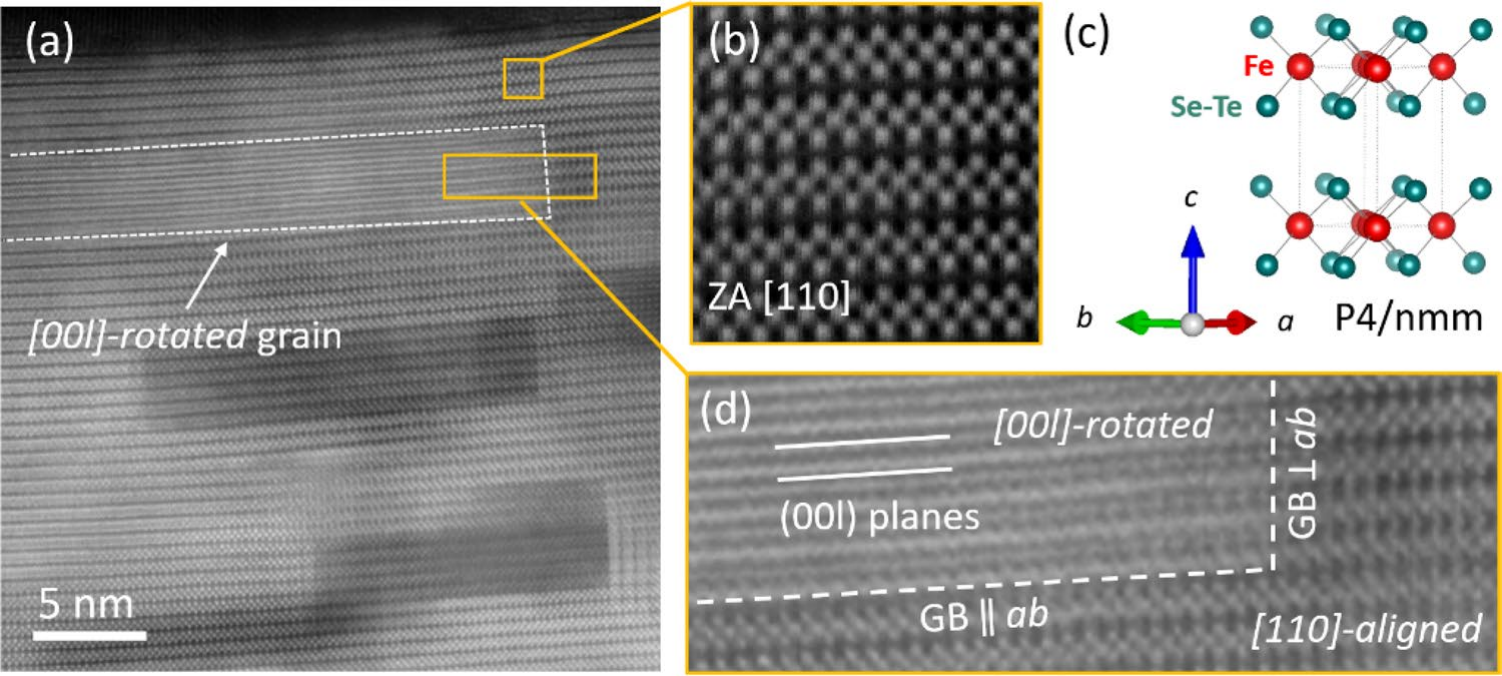
Structure of the Fe(Se,Te) film at high-resolution. (**a**) High-resolution Z-contrast STEM image of the [110]-oriented FeSe_1−x_Te_x_ film. (**b**) Enlarged view of the atomic lattice and corresponding 3D atomic model of the P4/nmm unit cell in (**c**). (**d**) Detail of grain boundaries between a rotated grain and a not-rotated one.

In order to investigate the atomic structure of the various grains that make up the film, we made cross-view STEM measurements with atomic resolution. From the high-resolution Z-contrast image in Fig. 3, it emerges that the grains are generally oriented along the [110] direction, ﻿‖ to [100]CaF_2_ substrate as obtained from SAED pattern in Fig. 1b. The crystal lattice belongs to the Pa/nmm tetragonal phase of FeSe_1−x_Te_x_. The different stoichiometry of grains is evidenced by the intensity modulation among grains. As a consequence, this generates a lattice mismatch between grains with different stoichiometry: this mismatch implies also that grains are not properly aligned with the main [110] direction due to the presence of crystal lattice distortions that accommodate the mismatch. Indeed, from electron diffraction measurements conducted on a cross-view sample, a 4º distortion of the lattice was recognized (see Supplementary Information Figure S1). In some grains, the atomic structure is not resolved and just (00*l*) planes emerge as indicated by white dotted line in Fig. 3a and in its enlarged view in Fig. 3b). This indicates that grains with other in-plane orientations are present together with the dominant *[110]-aligned* ones. These grains are misaligned by a rotation around the [00*l*] direction and we name them *[00l]-rotated* grains. The interface between a *[00l]-rotated* grain and a non-rotated one (*[110]-aligned*) determine two-dimensional defects at the grain boundaries (GB). These 2D defects can be essentially divided into two types, depending on whether they are at the “top and bottom” or at the “sides” of a *[00l]-rotated* grain: we call these defects “GB ‖ *ab*” and “GB ⟂ *ab*”, to remind that they extend on planes parallel and orthogonal to the *ab* plane respectively.

### Pinning properties

**Figure 4:**
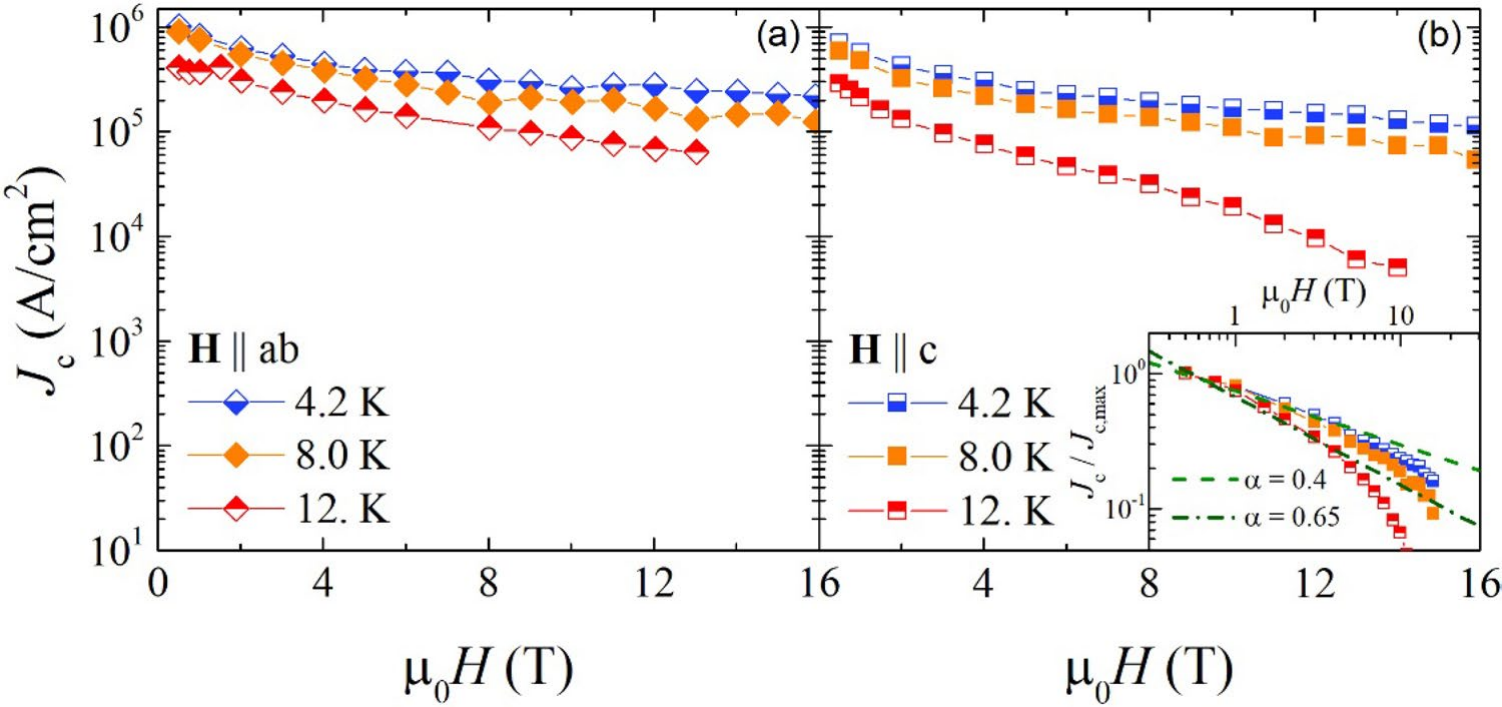
Critical current J_c_ as a function of magnetic field at different temperatures of 4.2, 8 and 12 K with field parallel (**a**) and perpendicular (**b**) to the *ab* planes. In the inset of panel b, the fitting curve based on the relation are J_c_(B) ∝ B^−α^ reported.

In Fig. 4, we report J_c_ as a function of the magnetic field up to 16 T at different temperatures of 4.2, 8 and 12 K with field parallel (a) and perpendicular (b) to the *ab* planes. TDGL based simulations have demonstrated that it is possible to retrieve information about the size of the pinning sites through an analysis of the J_c_(B) curves^13,14^. In particular, considering the relation J_c_(B) ∝ B^−α^, there is a dependence of the α exponent on the volume fraction of non-superconducting spherical inclusions. As reported in the inset of Fig. 4, the value of α is ≈ 0.65 for data at 12 K, while it is ≈ 0.4 for data at 4.2 K, and 8 K^13^. From these values we can infer the presence of large defects with size ≈ 4ξ for which it should be α ≈ 0.625, and/or of small defects with size ≈ 2ξ for which it should be α between 0.25 and 0.35 (ξ being the coherence length). The ambiguity in these results can rise from the highly non-symmetrical shape of the defects which have been identified (see Fig. 3), as well as from the presence of *ΔT*_c_-type pinning sites.

**Figure 5:**
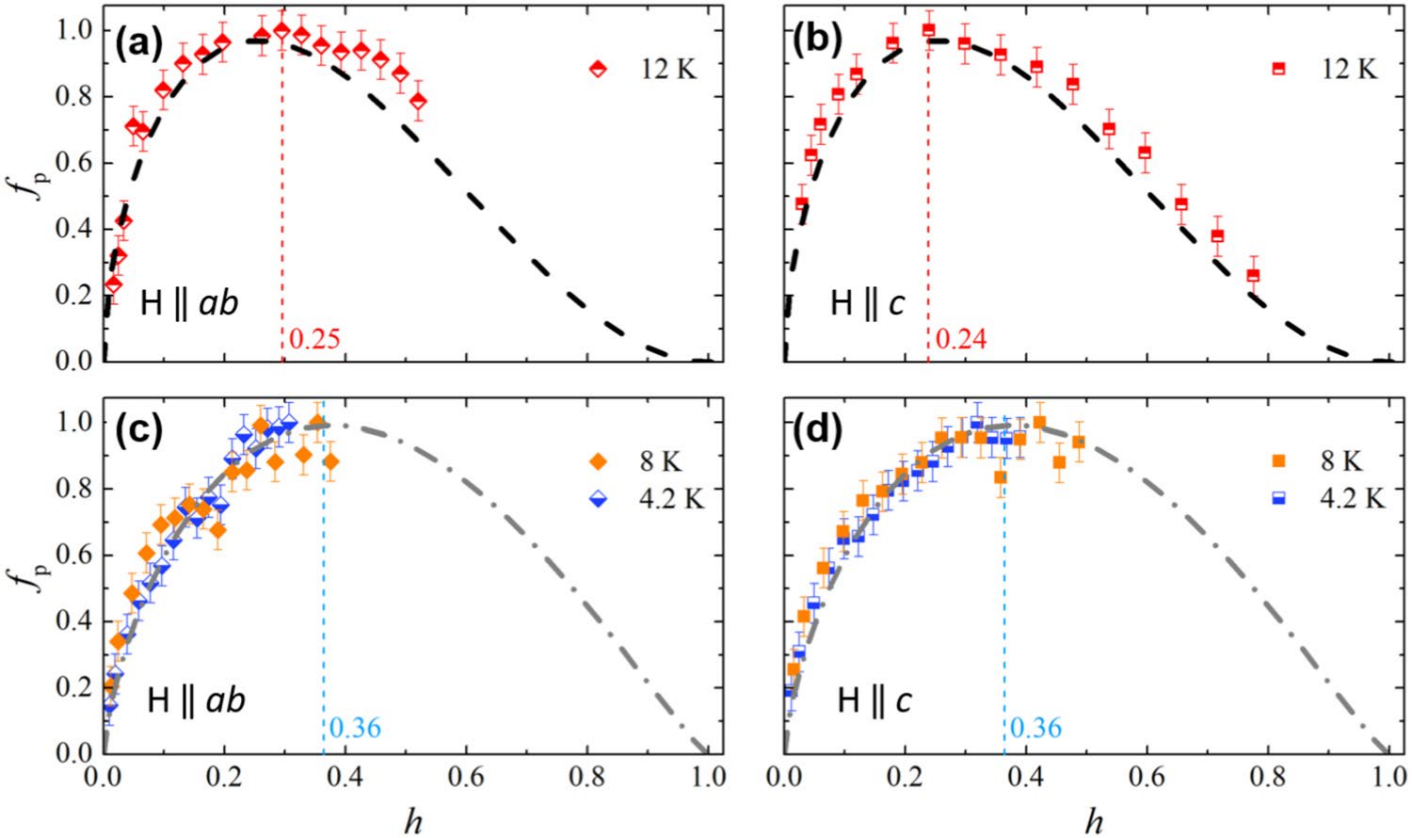
Normalized pinning force as a function of the reduced field h = H/H_irr_ at different temperatures of 4.2, 8 and 12 K, with field parallel (**a**, **c**) and perpendicular (**b**, **d**) to the *ab* planes. Dashed and dot-dashed lines come from Dew-Hughes fitting procedure.

In order to clarify the results obtained by the previous analysis, we extracted the pinning force f_p_ = J_c_xB from Jc measurements and we analyzed the resulting curves in the framework of the Dew-Hughes model. Indeed, the analysis of the pinning forces in the framework of Dew-Hughes model^15^ gives information on the nature and dimensionality of the pinning centers. In Fig. 5, we present the pinning force curves normalized to their maximum value, plotted as a function of the reduced field h = H/H_irr_. The normalizing field H_irr_ was estimated as the value at which the J_c_^1/2^(µ_0_H) curve is linearly extrapolated to zero.

The directions of the applied field is not influencing the pinning forces trend, as seen by comparing left and right panels in Fig. 5, where the position of the maximum pinning force at H‖*ab* and H‖*c* is shifted similarly from low (4.2, 8 K) to high (12 K) temperatures. This confirms a very weak anisotropy of the pinning mechanism in these samples.

The pinning force variation with temperature suggests that different pinning mechanisms are active in different temperature regimes, and these mechanisms can be identified using the Dew-Hughes fitting procedure. Hereafter, we use a fitting procedure based on this model which considers the possible presence of two main contributions to the material pinning. The procedure follows from the ansatz by Küpfer et al.*,* and its later development by Baumgartner et al.^16,17^. The validity of this empirical procedure is supported by the consistency of the pinning mechanisms that come out from the independent fits at the different temperatures and by the plausibility of these pinning mechanisms, given by the correspondence with the results of the structural and chemical analyses.

At the higher temperature of 12 K, i.e. close to T_c_, the data have been fitted by the relation *f*_*p*_ = *C*·*h*^*p*^(1 − *h*)^*q*^. The resulting values of the *C*, *p* and *q* parameters are not in agreement with any of the sets predicted by the canonical Dew-Hughes model. Thus, we identify the set of *C*, *p*, and *q* which gives the highest confidence with the experimental data, then we added a second term so that *f*_*p*_(*h*) = *w*·*f*_*p*,1_(*h*) + (1 − *w*)·*f*_*p*,2_(*h*). In this procedure, the relative weight of the two contributions *w* is the only fitting parameter and each *f*_*p*,*i*_ is expressed by the standard Dew-Hughes relation *f*_*p,i*_ = *C*·*h*^pi^ (1 − *h*)^*q*i^. It results *C*_1_ = 3.49, *p*_1_ = 0.5, *q*_1_ = 2 and *C*_2_ = 6.75, *p*_2_ = 1, *q*_2_ = 2, which according to Dew-Hughes model correspond to 2D *δl* and 1D *δl* pinning centers, respectively (black dashed line in the Fig. 5a, b). The resulting value for *w* is 0.68 ± 0.05, which reflects the presence of these two types of pinning centers, as previously reported^18^. Moreover, the values of the reduced field corresponding to the maximum pinning force, *h*_*max*_, are 0.24 and 0.25 for H‖*c* and H‖*ab*, respectively. These results are in perfect agreement with other previous results^9,18^, and with the presence of both 2D *δl* and 1D *δl* pinning centers in these films. At the lower temperatures of 4.2 K and 8 K, i.e. below 0.5 T_c_, we could describe the data within the Dew-Hughes model with a single pinning contribution. From the fit we get the values *C* = 3.4 ± 0.3, *p* = 0.71 ± 0.04, *q* = 1.17 ± 0.10 (grey dot-dashed line in the Fig. 5c, d). The *f*_*p*_ curve shape is slightly modified with respect to that at higher temperature, with a shift of the *h*_*max*_ value up to 0.36. This reflects an additional contribution of a *ΔT*_c_-type pinning, for which the Dew-Hughes model predicts *C* = 4, *p* = 1, *q* = 1^15^. It can be argued that this *ΔT*_c_-type pinning appearing at the lower temperatures (4.2 K and 8 K) becomes relatively less important at the highest temperature (12 K), where the 2D *δl-*type and 1D *δl*-type pinning contributions are dominant. However, even at 12 K the *ΔT*_c_-type pinning contribution can be identified from the shoulder in the experimental curves on the right-side of the maximum with respect to the fitting curves (see the right-side of the maximum in the Fig. 5a, b).

**Figure 6:**
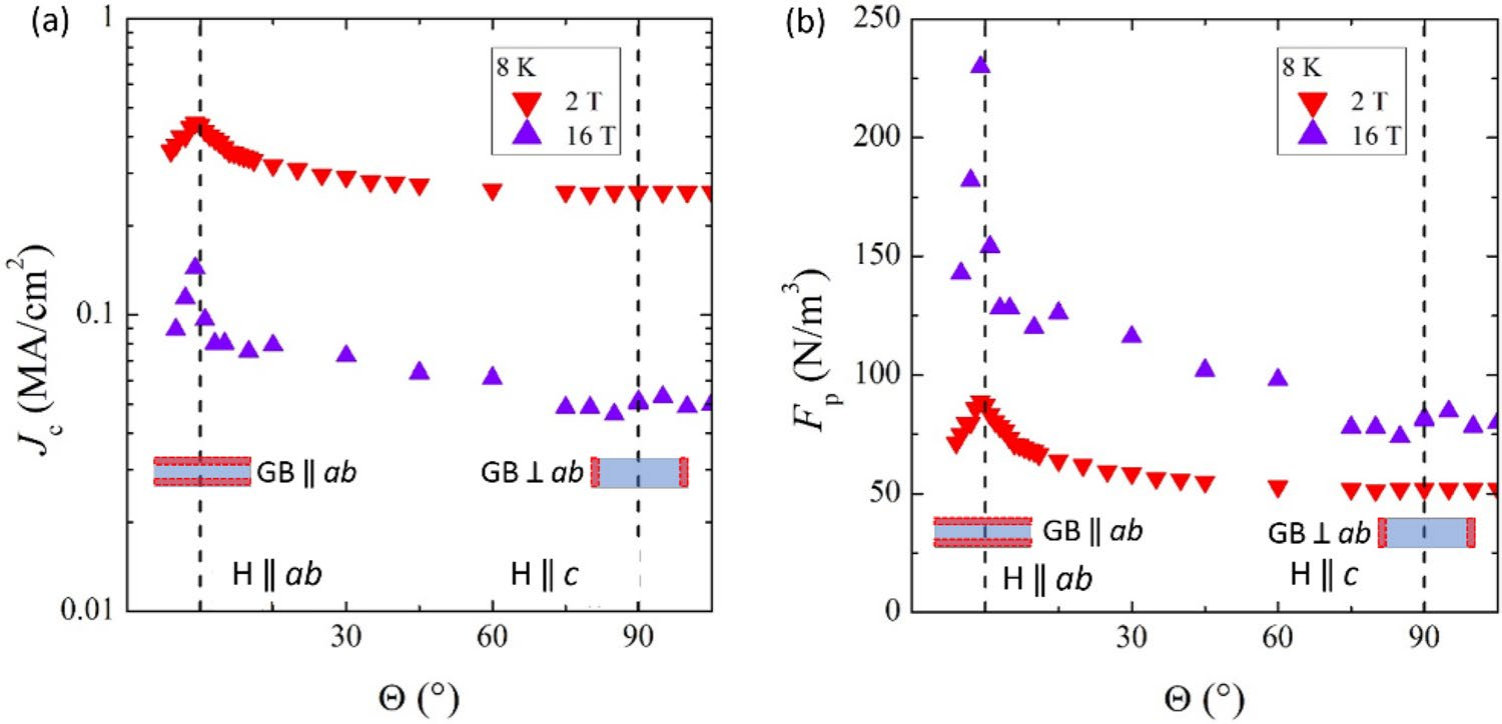
Angular dependence of the critical current density J_c_(θ) in semi-log scale (**a**) and pinning force (**b**), measured at 8 K and in fields of 2 and 16 T.

The full angular dependence of the critical current density J_c_(θ) measured at the fixed temperature of 8 K is shown in Fig. 6a, for fields of 2 and 16 T. The anisotropy factor γ_J_ = J_c_(θ = 0°)/J_c_(θ = 90°) is confirmed to be very low, between 2 and 3, at low and high applied magnetic fields, respectively. Moreover, a huge peak is observed for the orientation of the applied field in the *ab* plane of the superconducting film, i.e. θ = 0°, which is the signature of the pinning mechanism related to the layered microstructure of this material and to the 2D GB ‖ *ab* defects. At θ = 90° no peak is observed at 2 T, whereas a small peak is detectable at the highest field of 16 T, visible on the semi-log scale in Fig. 6a. This feature correlates with the 2D GB ⟂ *ab* observed defects, and indicates that *c*-axis correlated defects might become active as pinning centers at high fields. A wider peak related to *c*-axis pinning appearing above 3 T was also observed in Ref.^19^ in thin films grown on CaF_2_, as well as in Refs.^5,20^, although for the same material grown on a different substrate. In other cases, where no granular structure or large extended defects were observed throughout the whole film, only a very weak angular dependence was observed with increasing magnetic fields just for the *ab* parallel orientation of the applied field^21,22^.

**Figure 7:**
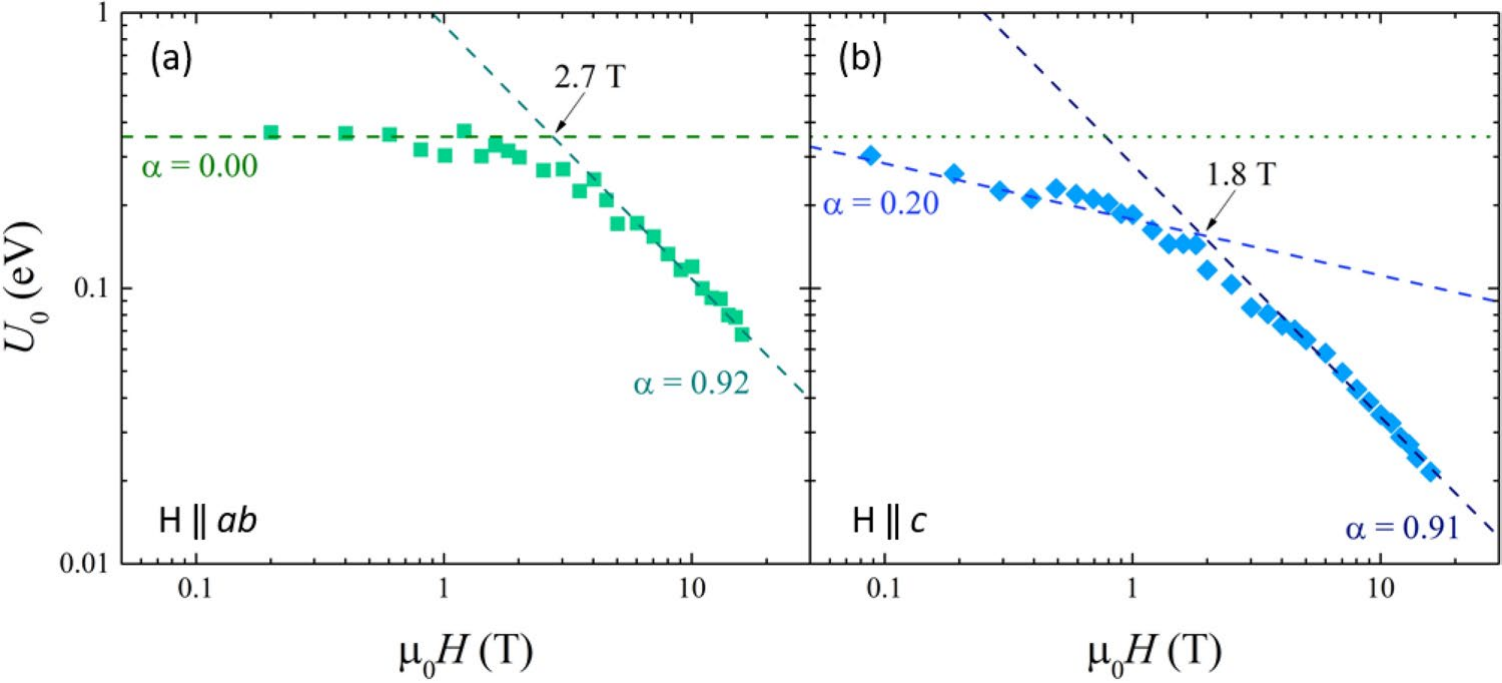
Activation pinning energy as a function of the magnetic field extracted from resistive transitions for field parallel (**a**) and perpendicular (**b**) to the *ab* planes.

The presence of such a smaller and spread *c*-axis correlated peak in the J_c_(θ) data, as well as in the corresponding pinning force F_p_(θ) curve shown in Fig. 6b, underlines the presence of a pinning mechanism that is becoming more effective at higher fields, suggesting that a cooperative interaction should become active at fields above a certain threshold. In order to clarify this point, the activation pinning energy was estimated as a function of the magnetic field intensity by the analysis of the Arrhenius plot of the resistance versus temperature curves. The activation pinning energy extracted from the resistive transitions is plotted as a function of the magnetic field in Fig. 7 and a threshold field is clearly identified below which a single-vortex like pinning regime is dominant, whereas a collective pinning regime is established above such threshold. The threshold field is around 3 T for the case of parallel magnetic field and 2 T for the perpendicular orientation.

Based on these findings, a correlation with the microstructure of the sample on a nanoscale can be established to clarify the nature of those defects more efficient at high magnetic fields.

## Discussion

The structural, microstructural and elemental characterization directly compared with electrical transport data of critical current density, pinning force and activation energy in the superconducting state allows us to formulate reasonable hypotheses to account for the enhanced pinning properties and low anisotropy of Fe(Se,Te) films grown on CaF_2_ substrates.

The dominant pinning mechanisms observed from the fitting of the pinning force curves (Fig. 5) are (1) *ΔT*_c_ pinning, dominating at lower temperatures 4.2–8 K and minor at 12 K, and (2) 2D *δl* and 1D *δl* pinning well identified only at the higher temperature 12 K, where the *ΔT*_c_ pinning mechanism is relatively less important, but possibly present at all temperatures. We suggest that the source of such pinning mechanisms are the grain boundaries of the regularly shaped rotated grains well visible in Figs. 1 and 3, as well as the lattice distortions and 2D defects identified in Fig. 3, and the high-mass walls shown in Fig. 2. These elongated and regularly-shaped grains and walls also have different Se:Te stoichiometry with respect to the adjacent grains (Figs. 1, 2), hence their boundaries may play multifold roles, namely 2D *δl* pinning and *ΔT*_c_ pinning, with a relative efficiency that depends on temperature.

The identification of the regularly shaped rotated grains, lattice distortions and 2D defects in Figs. 1 and 3 as the main pinning centers also accounts for the angular and field dependence of J_c_ displayed in Fig. 6a. Indeed, the large aspect ratio of the regularly shaped rotated grains provides strong pinning for in-plane H direction, because the vortex lines are pinned by the more extended planar grain boundaries (2D GB ‖ *ab* defects), thus accounting for the high J_c_ peak at θ = 0° visible at all fields in Fig. 6. On the other hand, for out-of-plane H, the smaller boundaries of the grains along the *c*-axis (2D GB ⟂ *ab* defects) are not as much efficient in pinning single vortex lines at low field. However, when the field increases and the inter-vortex interaction increases, the vortex lines can be collectively pinned by multiple 2D GB ⟂ *ab* defects and the pinning mechanism becomes more efficient, as visible from the small J_c_ peak at θ = 90° appearing at large field 16 T in Fig. 6a. This crossover from low to high field pinning regimes is also confirmed by the activation energy analysis displayed in Fig. 7, where threshold fields around 2–3 T are extracted, which correspond to inter-vortex distances of tens of nanometers and are comparable to the average distance between different grain boundaries along the *c*-axis.

It is worth nothing that the observed regularly shaped misoriented grains are only a minor fraction and they are not continuous throughout the film, thus the impact of these grain boundaries and associated weak-link effects on the critical current density are not expected to be significant^23^. It is also worth noting that the high-mass walls, although actually wall-like in shape, are made of superconducting Fe(Se,Te) with higher mass stoichiometry. Hence, however large their thickness is, they are no current blockers. Indeed, no significant decrease in J_c_ at low field is observed in our samples (see Fig. 4), which is also consistent with literature results on similar systems^24^.

## Conclusions

In this experimental study of epitaxial Fe(Se,Te) films grown on CaF_2_ substrates, we investigated structure, microstructure, stoichiometry, and superconducting electrical transport properties in magnetic fields with different directions and magnitudes, thus establishing a correlation between microstructural and compositional properties and pinning properties.

High-resolution STEM analyses indicate that the crystal lattice belongs to the Pa/nmm tetragonal phase of Fe(Se,Te), with dominant [110] in-plane orientation and other additional orientations also present in some rotated grains. STEM and EELS analyses reveal domains of elongated and almost rectangular shape, with typical 15–20 nm width along the ab planes and 3–5 nm height along the *c*-axis. These domains are characterized by different Se:Te stoichiometry, while Fe content is pretty uniform throughout the sample. Crystal lattice distortions and 2D defects are present at the grain boundaries.

Transport critical current density measurements show a weak anisotropy in the direction of magnetic field with respect to the crystalline axes. The analysis of the pinning force indicates that *ΔT*_c_-type pinning is dominant at the lower temperatures 4.2–8 K, while at 12 K this contribution becomes relatively weaker and two-dimensional (2D) *δl*-type and one-dimensional (1D) *δl*-type pinning mechanisms dominate. These mechanisms can be associated with the above mentioned compositional (*ΔT*_c_-type pinning) and microstructural (*δl*-type pinning) defects. The angular and field dependence of J_c_ shows a sharp peak for field oriented in-plane, which can be directly correlated to the extended planar grain boundaries of the elongated domains and the related 2D lattice distortions. A crossover field of 2–3 T is also identified from the analysis of pinning activation energy and from the appearance of a small peak in the angular dependence of J_c_ for field parallel to the *c*-axis, possibly marking a transition from a regime of single vortex pinning by microstructural defects to a regime of collective pinning.

In conclusion, the combination of high resolution lattice imaging and superconducting transport measurements in magnetic field allowed to gain deep understanding of the pinning mechanisms into play in Fe(Se,Te) films grown on CaF_2_. As these pinning mechanisms are mostly related to growth induced defects, they are specific of the considered Fe(Se,Te)/substrate system and, for this reason, the results of this work cannot be directly extended to Fe(Se,Te)-based coated conductors grown on technical substrates. However this approach can be applied to Fe(Se,Te)-based coated conductors and other superconducting systems developed for large scale applications, where understanding of the pinning mechanisms is the prerequisite for controlling and optimizing fabrication procedures.

## Methods

### Fe(Se,Te) thin film deposition

Fe(Se,Te) thin films of about 100 nm thickness were deposited on [001] CaF_2_ substrates in a high vacuum Pulsed Lased Deposition (PLD) system equipped with a Nd:YAG laser at 1024 nm. The FeSe_0.5_Te_0.5_ PLD target was synthesized with a two-step method^25^. The optimized laser parameters to obtain high quality epitaxial 11 thin films^9^ were 3 Hz repetition rate, 2 J/cm^2^ laser fluency (2 mm^2^ spot size) and 5 cm distance between target and sample. The deposition was carried out at a pressure of 10^−8^ mbar, while the substrate was kept at 350 °C.

### Electron microscopy characterization

A systematic chemical and microstructural characterization was conducted via TEM by using selected-area electron diffraction (SAED), STEM and EELS techniques. After the removal of the Fe(Se,Te) film from the CaF_2_ substrate by means of a mechanical liftoff process, cross-sectional TEM specimens were prepared through a conventional mechanical TEM sample preparation routine including cutting, gluing, grinding, polishing, followed by a final precision ion polishing by means of a GATAN PIPS II system. High angle annular dark-field scanning transmission electron microscopy (HAADF-STEM) was carried out on a probe Cs-corrected JEOL JEM-ARM200F, equipped with a cold FEG electron gun operated at 200 kV and a GIF Quantum ER energy filter by GATAN. All micrographs were acquired by using a probe convergence semi-angle of 33 mrad with the ADF detector collecting signals at a high inner semi-angle (80 mrad). Under these conditions the observed intensities in the STEM images are proportional to the atomic number Z^1.7^^26,27^ and the partial contributions from diffraction contrast and in-plane grain misorientation were minimized. EELS spectra were obtained at the electron transparent regions of the TEM specimen (t/λ = 0.8) with a spatial resolution of about 1 nm.

### Superconducting properties characterization

Superconducting measurements were carried out on a sample with a critical temperature T_c_ of 17.7 K, whose characterization is reported in Ref.^28^. In order to allow electrical transport measurements, the film was patterned through standard optical lithography, and the etching was performed by water-cooled argon ion milling (argon ion energy 500 eV). After the milling process, the photoresist was removed by mild sonication in acetone for a few tens of seconds and dried in nitrogen air. Nine Hall bar-shaped micro-bridges 20 μm wide and 50 μm long were realized^29^. The electrical transport properties were investigated by means of a Cryogenic Ltd. full cryogen free cryostat equipped with an integrated cryogen-free variable-temperature insert operating in the range 1.6–300 K up to a maximum magnetic field of 16 T. In this system, the sample was cooled by a continuous helium gas flow and the temperature stability was within 0.01 K. The electrical resistance measurements as a function of the temperature were performed by a four-probe method, and the critical current data were extracted from I-V measurements using the standard voltage criterion set at 10 µV/cm. The angular measurements were carried out by means of a double-axis rotator sample unit, always in a Lorentz-force configuration with the applied magnetic field perpendicular to the current flow. The rotation angle θ was determined by the applied magnetic field direction with respect to the *c*-axis of the films, so that θ = 0°corresponds to the field parallel to the surface of the film.

## Supplementary Information

Supplementary Figures.
