# Supplementary material for: Nanoscale analysis of superconducting Fe(Se,Te) epitaxial thin films and relationship with pinning properties
Source: Sci Rep. 2021 Oct 11;11:20100. doi: 10.1038/s41598-021-99574-5 (PMC8505440; doi:10.1038/s41598-021-99574-5)
Supplement: Supplementary file 1 — Supplementary Figures. [file 41598_2021_99574_MOESM1_ESM.docx]

**Nanoscale analysis of superconducting Fe(Se,Te) epitaxial thin films and relationship with pinning properties**

**Mario Scuderi^1*^, Ilaria Pallecchi^2*^, Antonio Leo^3,4^, Angela Nigro^3,4^, Gaia Grimaldi^3*^, Carlo Ferdeghini^2^, Corrado Spinella^1^, Marco Guidolin^5^, Antonio Trotta^5^, Valeria Braccini^2^**

^1^ CNR – IMM Catania Headquarter, Strada VIII n.5 Zona Industriale, Catania 95121, Italy

^2^ CNR – SPIN Genova, Corso Perrone n.24, Genova 16152, Italy

^3^ CNR – SPIN Salerno, Via Giovanni Paolo II n.132, Fisciano, Salerno 84084, Italy

^4^ Dip. di Fisica “E.R. Caianiello”, Univ. Di Salerno, Via Giovanni Paolo II n.132, Fisciano, Salerno 84084, Italy

^5^ Eni Venezia, Via delle Industrie n.39, Marghera 30175, Italy

**TEM Bright-Field and SAED analysis**

Selected area electron diffraction analysis reveals a crystal-lattice distortion, as evidenced by the two electron diffraction patterns of Figure S1(b) and (c) relative to the two regions indicated in Figure S1(a).


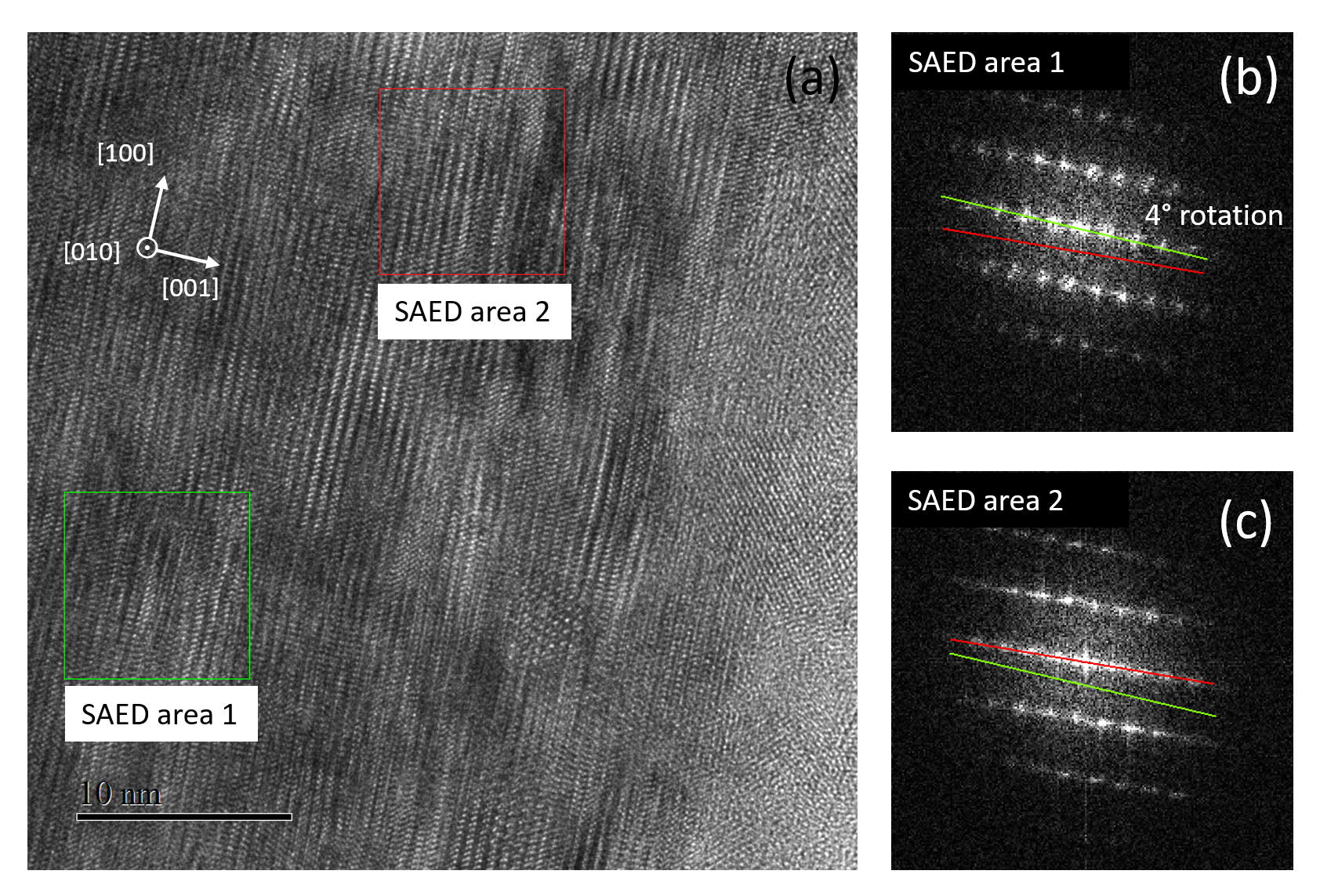


**Figure S1.** (a) TEM bright-field cross-view image of the Fe(Se,Te) film. (b) and (c) SAED of the two regions indicated in (a).

**EELS elemental mapping of Fe(Se,Te) plan-view**

Figure S2 shows a STEM dark-field plan-view of the Fe(Se,Te) and the relative EELS elemental maps obtained by selecting Fe L-edge, Se L-edge and Te M-edge. The bright contrast structures derive from two different causes: the ones with linear shape are the “high mass walls” while the other structures with an irregular shape are due to the thickening of the film in the areas where it is folded.

while the shape of the “high mass walls” are recognizable on the elemental maps as Fe-poor and Se-Te-rich structures, the thick region are not, Indeed, These latters are just thicker areas with the same stoichiometry as the film. In Figure S2(c) the elemental line scan showsthe raising of signal for the higher mass elements, namely Se and Te and the lack of signal for Fe, in correspondence to the high mass "wall”.


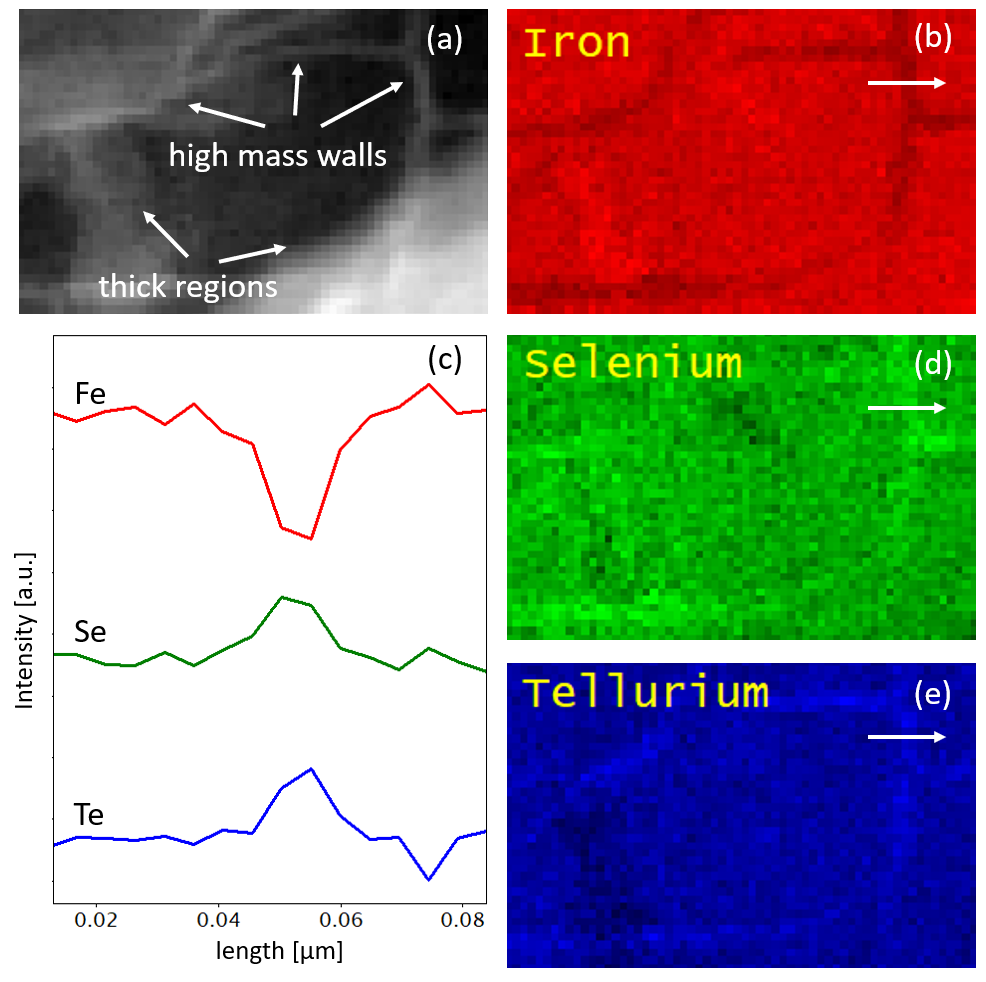


**Figure S2.** (a) STEM dark-field micrograph. The corresponding EELS Fe, Se and Te elemental maps are shown in (b), (d) and (e), respectively. (c) elemental line scan for Fe Se and Te crossing the high mass "walls” (white arrow in (b), (d) and (e) respectively).
